# Nursing highlights from the 2015 European Cancer Congress (ECCO18–ESMO40), 25–29 September 2015, Vienna: reinforcing multidisciplinarity

**DOI:** 10.3332/ecancer.2015.589

**Published:** 2015-11-05

**Authors:** Rosario Caruso, Danuta Lichosik

**Affiliations:** 1 Head of Health Professions Research and Development Unit, IRCCS Policlinico San Donato, Milan 20097, Italy; 2 Nurse Coordinator, IEOEDUCATION, School of Robotic Surgery, European Institute of Oncology, Milan 20141, Italy

**Keywords:** cancer, conference report, ECC2015, multidisciplinary approach, nursing

## Abstract

The 2015 European Cancer Congress (ECC2015) was the widest European platform for every professional involved in the struggle against cancer (17,656 participants, 2482 abstracts submitted). In this context, the nursing contribution was very relevant, involving high quality research and experience. The major nursing issues were: online information and support; innovations in cancer nursing roles; patient safety and the nursing workforce; managing older people with cancer and other co-morbidities; living with and beyond cancer; nutrition and body image changes; the changing face of cancer care for oncology nurses. Indeed, an important amount of research was presented during proffered papers and poster presentations by nurses from all over the world, concerning challenging issues, such as advanced nursing roles, end of life care, impact of cancer on patients and families, new developments, supportive and palliative care, survivorship and rehabilitation, symptom management and transitions in care.

Nurses’ presentations were mainly focused on understanding patients’ needs and on sharing the best evidence-based approach to meet those needs. This is particularly significant in a field where innovation develops rapidly in every area of clinical practice, such as cancer care, bridging the weaknesses between different approaches and profiles, within the paradigm of multidisciplinarity.

## Introduction

The 2015 European Cancer Congress (ECC2015) had the aim to reinforce the multidisciplinary approach in cancer care to a high degree of continuity with the 2013 European Cancer Congress [1]. ECC2015 was the widest European platform for every professional involved in the struggle against cancer under the different profiles needed to best face the diverse nuances around oncology, such as disease-specific professionals, scientists, patient advocates, and any oncology stakeholders. ECC2015 was directed to a global audience, and it was characterised by a very significant number of high-quality presentations, covering the main important areas of scientific research. The multidisciplinary breadth of ECC2015 was considerable starting from the programme, considering that many sessions were integrated between the different involved societies, bringing a productive dialogue and debate. This approach is the pillar of a modern paradigm where knowledge creation arises from a ‘team science’, as highlighted by John Yarlnord, European Cancer Organisation (ECCO) OncoPost editor-in-chief.

In this context, the nursing contribution was very relevant considering that the European Oncology Nursing Society (EONS) played a key role in supporting and developing European cancer nurses, and it was at the forefront of ECC2015 planning, proposing nursing and integrated sessions at the highest scientific levels. The importance of cancer nursing was well-highlighted by Professor Martine Piccart—Congress Chair and ECCO President—during the opening ceremony, when she stated that Oncology nursing across Europe is ECCO’s first priority because of the fact that an efficient nursing delivery of care could make a difference and add value to the treatment of cancer patients.

During the conference, major issues that were highlighted included [2]:

- Online information and support: benefits and risks
- Innovations in cancer nursing roles: learning from each other
- Patient safety and the nursing workforce: issues for cancer nursing
- Managing older people with cancer and other co-morbidities: an increasing challenge
- Living with and beyond cancer
- Nutrition and body image changes—a concern for all patients with cancer
- The changing face of cancer care for oncology nurses: the rising demand being placed on cancer services
- Proffered papers
- Posters

## News since ECC2015

The conference held in Vienna from the 25–29 September 2015, began with the opening ceremony where Professor Christoph Zielinski—Chair of the National Organising Committee—opened ECC2015 in front of hundreds of delegates. During the ceremony, Professor Piccart welcomed the participants, highlighting certain numbers representing the extent of the event: 17,656 participants, 2482 abstracts submitted (2023 accepted). She also discussed important issues concerning the changing face of cancer care and research, identifying which priorities ECCO have to face to advance in the care of cancer patients and in the development of onco-policy. The first priority was oncology nursing to interlink the diverse areas around oncology. Important topics concerning effective cancer treatments and new funding models were brought forth by Dr Margaret Foti, Chief Executive Officer of the American Association for Cancer Research.

During ECC2015, the EONS General Meeting took place on 26 September where the EONS members voted to approve the results of the recent EONS Executive Board elections and also where the two selected winners for the 2015 Novice Research Dissemination Award were nominated. They were Dr C Chabrera from Spain–Tecnocampus, Pompeu Fabra University Nursing, Mataro, with the abstract titled *‘A decision aid to support informed choices for patients recently diagnosed with prostate cancer: A randomised controlled trial’*, and Ms V Biagioli from Italy, University of Rome ‘Tor Vergata’, with the abstract titled *‘Propolis for prevention of chemo-induced oral mucositis in breast cancer patients: A randomised controlled trial’*.

The ECC2015 Nursing Track who had the goal of exploring the complexity involved in the daily practice in cancer care, highlighted on innovations and future perspective. According to the EONS policy, there is a convergence of views in considering which are the pillars of cancer care:

1. person-centred approach
2. evidence-based practice, supported by research
3. delivery of care in a systematic manner
4. delivery of care in a quality practice environment
5. meeting and understanding society’s needs

## Online information and support: benefits and risks

This session chaired by A Margulies (Switzerland) began with a lecture entitled *‘The role of patient experiences as an online resource’*, and the speaker was L Carrasqueiro from the United Kingdom. The presentation was aimed at better understanding the role of the internet as a source of others’ experiences of health and illness. In this way, nurses have to be aware about the value of patient experiences to other patients. Next Verdonck-de Leeuw (Netherlands)—with a lecture titled *‘Benefits and risks of e-health information sources for people living with cancer’*—emphasised on the potentiality of eHealth to improve the efficiency of supportive care and improve patient empowerment, and to support self-management strategies derived from tailored information to best meet every patient’s need.

## Innovations in cancer nursing roles: learning from each other

This session was chaired by K Lokar (Slovenia) and T Wiseman (United Kingdom), and it was characterised by the sharing of experiences and studies of nurses who achieved grants and awards. C Oakley (United Kingdom)—with a presentation titled *‘Extended roles in oncology nursing’*—had aimed at more thoroughly understanding the advanced practices, the role of nurse as a consultant role, and the extention of a nurse’s consultant role taking into account the challenges and the opportunities. Next M Eicher (Switzerland) defined the meaning of resilience in adult cancer care and its association with supportive care needs, also describing the development of a supportive care-intervention based on electronic assessment of resilience and unmet supportive care needs, direct feedback, and tailored intervention proposition for nurses and oncologists in outpatient cancer care. Subsequently, F Maddalena (Belgium) explained the changes in the method of cancer treatments stating that oral therapies are in constant growth. She also shared the experience of two specialised centres in France that have developed a therapeutic education programme and several learning points. Further, P Stolz Baskett (Switzerland) presented his research visit undertaken as part of the EONS Research Travel Grant Award 2014 to the Peter MacCallum Cancer Centre in Melbourne, Australia. In the same way, A Harrow (United Kingdom) described the rationale for applying for EONS research travel grant and also shared her experience.

## Patient safety and the nursing workforce: issues for cancer nursing

This session was chaired by the EONS President, Professor Daniel Kelly, and the session addressed issues deeply felt by cancer nurses. The first speaker, J Maben (United Kingdom), described the importance of staff well being and patient experience to ensure high quality and safety in the standards of care. After that, D L Schwappach (Switzerland) described the patients’ and the nurses’ perspectives on patient safety. Another important insight was given by A Jones (United Kingdom), focusing on the nature and scale of harm that occurs within healthcare, and how other industries and sectors have responded to the challenge of maintaining employee and customer safety.

There is important evidence that shows how the nursing workforce is linked to patient safety and outcomes. The nursing workforce is the key element in determining the quality of care, patient safety, and the best outcomes for patients. Indeed, nurse staffing is a crucial health policy issue, and there is a great deal of consensus among researchers in considering the significant relationship between nurses and best outcomes. However, it seems that awareness regarding this relationship is weak among the same nurses and especially for those in nursing management.

## Managing older people with cancer and other co-morbidities: an increasing challenge

This session was chaired by P Crombez (Belgium) and Professor L Sharp (Sweden). The first speaker, G Catania (Italy) talked about new models of follow-up for increasingly complex patient profiles and also focusing on the description of the cancer survivors needs. According to Catania, cancer survivors require care to be tailored to their precise needs, where the nurses play an important role in the shaping and developing of the new follow-up models. Next the presentation of F Van den Berkmortel (Netherlands) aimed to describe how complex the care needs in the clinic can be by bringing up the example of polypharmacy and its consequences in geriatric oncology. Van den Berkmortel also described which tools are useful to measure and treat polypharmacy. Subsequent to that, C Kenis (Belgium) emphasised the importance of appropriate assessment for older people with cancer, focusing on the geriatric screening and assessment, and giving practical recommendations. The last speaker of this session was I Rahm Hallberg (Sweden) with a presentation aimed at discussing and describing the ageing process, functional ability, and quality of life (QOL) in the different age-brackets. Furthermore, older peoples’ QOL and the impact from cancer and cancer treatment were discussed by Hallberg, in particular describing the impact of resilience and social resources.

The issue of older cancer patients poses an increasing problem to address. This subject was also discussed in the Oncopolicy Forum, where Professor R Audisio—European Society of Surgical Oncology (ESSO) President—stated that the time bomb of cancer in the elderly patient is real, considering that within 15 years, 12 million people (mostly over 65 years old) will die from neoplastic diseases on an annual basis. The cancer care of senior patients involve some drawbacks, such as less capability to use online information, to be less aware about the treatment options, and difficulty to manage themselves during the stages of the disease. In this field, the multidisciplinary approach is the best way to meet oncogeriatric needs.

## Living with and beyond cancer

This special session was chaired by EONS Past-President, E van Muilekom (Netherlands). The first speaker was Z Maravic Stokic (United Kingdom) with a presentation aimed at describing the patient perspective of the benefits of physical activity during and following cancer treatment. The individual motivation during the treatment is described as a key component to achieve the best results following the same treatment. Another interesting issue—aimed at understanding the needs of families with/or at risk of having genetic mutations predisposing to breast and ovarian cancer—was presented by M Katapodi (Switzerland). It also presented a public health approach to identify and support families at risk of having genetic mutations predisposing to breast and ovarian cancer. Afterwards, G Ozakinci (United Kingdom) presented the issue of ‘the fear of cancer recurrence’. The literature shows a relationship among fears of recurrence and other health related outcomes, for this reason a psychological intervention for fears of cancer recurrence is very important.

## Nutrition and body image changes—a concern for all patients with cancer

This special session was chaired by MC Lacerda (Portugal). During the first presentation, Y Wengström (Sweden) highlighted the main issues for cancer practice concerning weight gain during cancer therapy. Weight gain could be an insidious enemy to prevent and address, considering that it could be very harmful for the motivation and mood of cancer patients. After that, professor M Wells (United Kingdom) assessed swallowing difficulties in head and neck cancer. She discussed the nature, experience, and prevalence of swallowing problems thereby highlighting the current evidence for their assessment and management. The last speaker of this session was F Strasser (Switzerland). He focused on his experience of the assessment and treatment of cancer cachexia. Many cancer patients have progressive weight loss, anorexia, and persistent erosion of host body cell mass in response to a malignant growth, going towards the cachexia syndrome. Dr Strasser shared a lot of useful indications to best deal with this syndrome in an evidence-based manner. This session was characterised by a thorough debate among the audience and speakers, because of the fact that nutrition and body image changes are issues that cross all oncological clinical fields.

## The changing face of cancer care for oncology nurses: the rising demand being placed on cancer services

This scientific symposium was chaired by B Grube (Denmark) and K Lokar (Slovenia). The first speaker was Professor C Foster (United Kingdom) with a presentation titled *‘Meeting the support needs of patients, carers & families after cancer treatment’*. Cancer care has important implications for patients and their families, and a supported self-management is needed to face the high complexity of cancer care as well as the challenges of cancer survivors. Next R Caruso (Italy) lectured on the factors underpinning workplace stress in cancer caring.

This issue was deeply felt by oncology nurses because of being faced daily with high demands. During the presentation, Caruso highlighted the main factors underpinning the workplace stress and eventually summarising the main evidences to support a preventive stress management. Many interesting reflections were shared during the debate, such as the difficulty of managing stress and being supported by an appropriate intervention when the stress threshold is exceeded. Next B Thoft Jensen (Denmark) shared the results of a trial, regarding a multidisciplinary rehabilitation in advanced bladder cancer. Her study was aimed at testing a multidisciplinary rehabilitation using an educational intervention. The session ended with the presentation of C Bailey (United Kingdom) aimed at describing three studies concerning emergency cancer admissions. She focused on the patients’ decision-making prior to an emergency admission, and described an interesting conceptual framework that could be useful to better understand what is behind the choice to head towards an emergency room.

## Proffered papers

The first session of proffered papers was chaired by T Wiseman (United Kingdom) and D Kelly (United Kingdom). In the first presented study, M Reaney (United Kingdom)—with a presentation entitled *‘Similarities and differences between symptoms and impacts of ovarian cancer as reported by the patients and their caregivers’*—showed how the symptom burden of ovarian cancer is significant for both patient and caregivers. Discrepancies between experienced and observed symptoms highlight the importance of directly capturing the patient perspective in clinical research. Subsequently another qualitative study was presented by S Williamson (United Kingdom). She presented the first UK study to explore the experiences of gay men with prostate cancer, and identified unmet needs unique to gay men diagnosed with prostate cancer which have implications for clinical practice, health policy and groups which provide support to men with prostate cancer. The third presentation (S Decosterd, Switzerland) was on the translation and validation of an instrument to measure oncology inpatient acuity from English into French. Next E Decoene (Belgium) presented a study on the factors influencing the process of medication (non-)adherence and (non-) persistence in breast cancer patients with adjuvant anti-hormonal therapy using a qualitative methodology. Interventions such as a multidisciplinary follow-up clinic, a nurse led clinic, patient support groups, information sessions, and brochures can be developed based on the results of that study. Another interesting experience was presented by AM Flores (USA), concerning the lymphoedema signs, symptoms, self-reported diagnosis, and referral to physical therapy among African American and low-income breast cancer survivors. Later AF Leclerc (Belgium) presented a study on the feasibility and psychological benefits of a multidisciplinary oncological rehabilitation programme in women after their treatments for breast cancer. The last speaker of the first proffered papers session was M Bendiane (France), presenting a case-control study on the analgesics use of French cancer survivors during the two first years after diagnosis. That study showed how the occurrence of pain among cancer survivors is frequent during the two first years after diagnosis but there are differences in the medical prescription of analgesics based on the patient’s gender, age, or social deprivation suggesting the existence of social barriers to pain management.

The second session of proffered papers was chaired by M Wells (United Kingdom) and M van Nijen (Netherlands). The first study of this session—presented by ML Mollerberg (Sweden)—was a population-based register study of cancer in Sweden. It was aimed at studying the effects of cancer diagnosis on the health of the patient’s partner. The partner’s risk of decreased health varied in relation to the type of the patient’s cancer as well as the severity and prognosis of that cancer. The second study—presented by A Arber (United Kingdom)—was a descriptive qualitative research to identify the awareness of family needs and the needs of dependent children of a parent with advanced cancer. According to that study, specialist staff require more support and guidance on assessing family needs regarding dependent children throughout the cancer trajectory. The third study—presented by S Williamson (United Kingdom)—was a non-inferiority randomised controlled with two arms: standard hospital follow-up and nurse-led telephone follow-up. There were no significant variation in satisfaction with information or the service between arms, and the levels of satisfaction were high. Next J Timmerman (Netherlands) presented a qualitative research to report the process of co-creation of an Information and Communication Technology (ICT)-supported cancer rehabilitation programme with and for lung cancer patients and their healthcare professionals. After that, C Olsson (Sweden) presented a study aimed at describing and exploring changes in sexuality, body image, and QOL in patients treated for haematologic malignancies from baseline until 12 months after treatment. The results showed how sexuality, body image, and QOL became negatively affected in patients with haematologic malignancies 45 years and older during treatment. Improvements were gradually seen after treatment, but the scores regarding sexual relationship were still affected after one year. Although sexual interest was reported to be low, this finding highlights the need of support to these patients regarding sexuality during follow-up care. The last research presented in this session was a pilot study to evaluate a screening-based nurse navigation intervention using a randomised controlled design. It was presented by BG Mertz (Denmark), and it will provide knowledge on the effectiveness of systematic symptom screening combined with nurse navigation, and it may contribute to future development of rehabilitation programmes.

## Posters

During ECC2015 an important number of posters were displayed covering the most challenging areas of cancer nursing practice, such as advanced nursing roles, end of life care, impact of cancer on patients and families, new developments, supportive and palliative care, survivorship and rehabilitation, symptom management and transitions in care. Many posters were discussed in discussion sessions, characterised by interesting debates where participants shared their experiences and strengthened their networks. Poster presentations have been shown to be a high quality moment of knowledge exchange.

**Figure 1.**
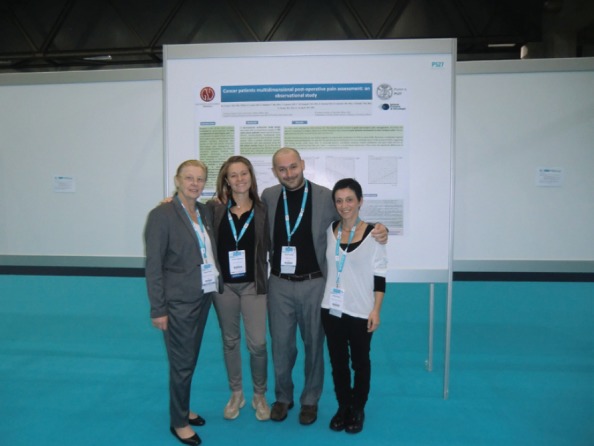
Italian delegates at ECC2015.

Figure 1 includes some Italian delegates in the poster area; from the right side, Debora Negri (European Institute of Oncology), Rosario Caruso (Policlinic San Donato), Federica Dellafiore (Policlinic San Donato), and Danuta Lichosik (European Institute of Oncology).

## Conclusion

ECC2015 was a high quality conference aimed at improving cancer patient outcomes through multidisciplinarity. In this context, cancer nursing has a recognised strategic role in a period of a diffuse sense of uncertainty, arising mainly from the expectations around innovations in cancer treatments. The best way to celebrate major discoveries in cancer care is to keep research alive in every area of oncology, including cancer nursing research. Efficient cancer nursing could enhance every patient outcome and it could be useful in reducing the gap between innovations and everyday practice, applying, and developing evidence-based models to deliver care.

ECC2015 was also a unique occasion to gain a deeper understanding of how the nursing workforce and roles are important in dealing with an increasing number of cancer patients and myriad therapeutic options. Nurses are focused on meeting patients’ needs, and this is particularly significant in a field where innovation develops rapidly in every area of clinical practice such as cancer care, where bridging the weaknesses between different approaches and profiles is done under the paradigm of multidisciplinarity.
